# Iatrogenic cerebral amyloid angiopathy: more cases to come

**DOI:** 10.3389/fneur.2025.1741885

**Published:** 2025-12-15

**Authors:** Till Turloff, Ulf Jensen-Kondering

**Affiliations:** Departement of Neuroradiology, Universitätsklinikum Schleswig-Holstein (UKSH) Campus Lübeck, Lübeck, Germany

**Keywords:** Lyodura^Ⓡ^, cadaveric dura mater, iatrogenic Creutzfeldt–Jakob disease (iCJD), iatrogenic cerebral amyloid angiopathy (iCAA), intracerebral hemorrhage

## 1 Introduction

Lyodura^Ⓡ^ was a commercially available cadaveric dura mater patch that was marketed worldwide by B. Braun Melsungen AG/Germany from 1969 to 1996 and widely used in neurosurgery for dural repair. Since 1987, it has been incriminated as a main agent of iatrogenic Creutzfeldt–Jakob disease (iCJD) by transmission of prions (1). More recently, it has also been associated with iatrogenic cerebral amyloid angiopathy (iCAA) by transmission of fibrillary amyloid aggregates (2). The mechanism with which the disease unfolds is unclear but is believed to occur in a prion like manner. Misfolded proteopathic seeds are believed to induce misfolding in other cells which in turn are also able to induce protein misfolding, thus reaching distant parts of the brain. After this first incubation phase, a second plateau phase follows during which neurotoxicity and clinical events occur (3). Its clinical and neuroradiological manifestations include stroke, seizures, transient focal neurological episodes, cognitive impairment, intraparenchymal and subarachnoid hemorrhages, cerebral microbleeds, and cortical superficial siderosis (4). iCAA may serve as a model disease to provide insights into sporadic CAA.

## 2 Discussion

To date, no certain numbers of cadaveric dura mater use were available, which precludes reliable risk assessment. It has been estimated, though, for Japan, Australia, South Korea, and more vaguely for the USA. For unknown reasons, its usage has been regionally diverse. In Japan, app. 20.000 patches have been used annually in the 1980s while in the USA < 10% of app. 4000 patches have been used annually (Table 1). Pikija et al. recently reported the numbers for the pediatric population from a single institution in central Europe. Over a period of 25 years, 34 patients were definitely exposed to Lyodura^Ⓡ^ during neurosurgical procedures. The authors report an incidence rate as high as 12% (5) for iCAA, which is in stark contrast to the incidence of iCJD after the use of cadaveric dura mater (Table 1). The authors state that for a number of reasons they most likely underestimated the true incidence: (1) MRI which might uncover subtle signs of CAA not visible on CT is not globally available and historically a young imaging modality. (2) Patients displaying only mild or clinically barely noticeable symptoms might not be picked up. (3) Many patients may have been misclassified as sporadic CAA and (4) many patients may have been lost to follow-up.

**Table 1:** Estimated Lyodura^Ⓡ^ use per country, reported cases, and incidences for iCJD and iCAA.

**Number of cases, Incidence**	**USA**	**Australia**	**Korea**	**Japan**
Estimated Lyodura^Ⓡ^ use, *n*	< 100/year (17)	~2,208–2,478 (18)	~57.434 (19)	~200.000 (20)
iCJD cases (confirmed Lyodura^Ⓡ^), *n*	4 (15)	5 (15)	2 (15)	142 (15)
iCJD incidence (confirmed Lyodura^Ⓡ^)	NA	0.23–0.43% (18)	0.02–0.09% (19)	0.03% (15)
iCAA cases (confirmed Lyodura^Ⓡ^), *n*	2 (21)	1 (22)	0	4 (23–26)

In addition, several aspects may indicate that the number of patients at risk may be still be higher: (1) Although the authors did not find a case of iCAA associated with other sources of cadaveric dura mater, other brands of cadaveric dura mater (6) and locally grafted cadaveric dura mater (7) have been implicated with the occurrence of iCJD. It is conceivable that these kinds of grafts were also a source of amyloid transmission. (2) Further, lyophilized cadaveric dura mater has been used in other procedures notably as an embolization agent in head and neck applications (8). (3) Other routes of infections (contaminated neurosurgical instruments (9), blood transfusion (10, 11)) also seem possible but unproven. (4) While the spectrum of underlying conditions leading to cadaveric dura mater use in the cohort described by Pikija et al. roughly matches Japanese patients treated with Lyodura^Ⓡ^ who developed iCJD (12) (tumor surgery and hemorrhage/trauma being the most common) Japanese patients' age was way outside the pediatric spectrum (mean age at surgery 44 years). However, a 20-year-old having received a cadaveric dura mater patch in 1995 may likely still be alive today and even be considered for the diagnosis of iCAA (4).

Although the authors did not find a case of iCAA associated with other sources of cadaveric dura mater, other brands of cadaveric dura mater (6) and locally grafted cadaveric dura mater (7) have been implicated with the occurrence of iCJD. It is conceivable that these kinds of grafts were also a source of amyloid transmission. Further, lyophilized cadaveric dura mater has been used in other procedures, notably as an embolization agent in head-and-neck applications (8). Other routes of infection [contaminated neurosurgical instruments (9) and blood transfusion (10, 11)] also seem possible but unproven.

While the spectrum of underlying conditions leading to cadaveric dura mater use in the cohort described by Pikija et al. roughly matches Japanese patients treated with Lyodura^Ⓡ^ who developed iCJD (12) (tumor surgery and hemorrhage/trauma being the most common), Japanese patients' age was way outside the pediatric spectrum (mean age at surgery: 44 years). However, a 20-year-old patient having received a cadaveric dura mater patch in 1995 may potentially still be alive today and even be considered for the diagnosis of iCAA (4). As for now, iCAA cannot reliably be differentiated from sporadic CAA (besides age of onset and history of exposure). Although preliminary, p-tau levels in CSF may be used for differentiation (13). Further, there is evidence that some hemorrhagic MRI markers occur in a centrifugal manner from the site of surgery (4, 14).

The mean incubation period for iCJD is 12 years (1.3–30 years) (15). The latency period for iCAA is reported to be much longer (mean: 34 years, range: 25–46 years) (4). A broad peak of occurrence of iCJD was reported in the mid-to-late 1990s and early 2000s (15). If the latency for iCAA is superimposed, a peak could be expected in the 2020s.

The last reported number of patients included in the international registry for iCAA, the largest collection of patients with iCAA so far, is 88 (16). This indicates that the gap between expected and reported cases is still wide (Table 1). Neuroradiologists, neurologists, and neurosurgeons have to identify the remaining patients.
